# Salt Preference is Linked to Hypertension and not to Aging

**DOI:** 10.5935/abc.20190157

**Published:** 2019-09

**Authors:** Patrícia Teixeira Meirelles Villela, Eduardo Borges de-Oliveira, Paula Teixeira Meirelles Villela, Jose Maria Thiago Bonardi, Rodrigo Fenner Bertani, Julio Cesar Moriguti, Eduardo Ferriolli, Nereida K. C. Lima

**Affiliations:** Faculdade de Medicina de Ribeirão Preto - Universidade de São Paulo (FMRP-USP) - Divisão de Clínica Médica Geral e Geriatria, Ribeirão Preto, SP - Brazil

**Keywords:** Aged, Aging, Salt Tolerance, Food Preferences, Sodium Chloride,Dietary/adverse effects, Flavoring Agents

## Abstract

**Background:**

Seasoning is one of the recommended strategies to reduce salt in foods. However, only a few studies have studied salt preference changes using seasoning.

**Objectives:**

The aim of this study was to compare preference for salty bread, and if seasoning can change preference in hypertensive and normotensive, young and older outpatients.

**Methods:**

Outpatients (n = 118) were classified in four groups: older hypertensive subjects (OH) (n = 32), young hypertensive (YH) (n = 25); older normotensive individuals (ON) (n = 28), and young normotensive (YN) (n = 33). First, volunteers random tasted bread samples with three different salt concentrations. After two weeks, they tasted the same types of breads, with seasoning added in all. Blood pressure (BP), 24-hour urinary sodium and potassium excretion (UNaV, UKV) were measured twice. Analysis: Fisher exact test, McNamer’s test and ANCOVA. Statistical significance: p < 0.05.

**Results:**

Systolic BP, UNaV, and UKV were greater in HO and HY and they had a higher preference for saltier samples than normotensive groups (HO: 71.9%, HY: 56% vs. NO: 25%, NY; 6%, p<0.01). With oregano, hypertensive individuals preferred smaller concentrations of salt, with reduced choice for saltier samples (HO: 71.9% to 21.9%, and HY: 56% to 16%, p = 0.02), NO preferred the lowest salt concentration sample (53.6% vs. 14.3%, p < 0.01), and NY further increased the preference for the lowest one (63.6% vs. 39.4%, p = 0.03).

**Conclusions:**

Older and younger hypertensive individuals prefer and consume more salt than normotensive ones, and the seasoned bread induced all groups to choose food with less salt. Salt preference is linked to hypertension and not to aging in outpatients.

## Introduction

Although the relationship between high salt intake and hypertension is well established^1-4^ and salt consumption by the world population is known to be higher than recommended,^4-7^ only a few studies have assessed the preference for salty foods and studied the preference changes using seasoning among hypertensive and normotensive individuals.^8,9^ Many countries are adopting different strategies to reduce salt intake by the population worldwide. The current public health recommendations in most countries are to reduce salt intake from about 9-12 g/day to 5-6 g/day.^10-12^

The 2010 World Health Organizations (WHO)^3^ global status report on non- communicable diseases urged member states to take immediate actions to reduce salt intake. To this end, the WHO recommended a 30% reduction in salt intake by 2025, with an eventual target of 5 g per day for adults and lower levels for children based on calorie intake.^13^

Sodium chloride is added to processed foods for palatability, preservation and processing reasons.^14,15^

One of the recommended steps to lower salt intake by the Food and Drugs Administration^16^ is to flavor food with pepper and other herbs and spices instead of salt.

Villela et al.^9^ compared the preference for salty foods between elderly hypertensive and normotensive subjects and showed that hypertensive individuals prefer and consume more salty foods than normotensive individuals. Some studies have shown that older people prefer more pronounced flavors than young people since the number of papillae and taste buds decreases with age.^17,18^

The INTERSALT study^19^ suggested a strong relation between salt intake and a progressive increase in blood pressure (BP) with age up to 4 mm Hg per year for a 6 g/day salt intake. A reduction in salt intake is therefore likely to attenuate the rise of BP with aging, in addition to having an immediate BP lowering effect.^20^

Since raised BP throughout its range is a major cause of cardiovascular disease, a reduction in salt intake, if it lowered BP, would reduce cardiovascular risk.^1^ Scientific research on this topic is scarce and only a limited number of studies have been performed in an experimental real-life setting.^14^

The aim of the present study was to compare the preference for salty foods among elderly and young individuals and hypertensive and normotensive ones and to determine if seasoning food can change the preference for salt. Another aim was to assess the habitual consumption of sodium and potassium, as well as BP and body mass index (BMI) in the different groups.

## Methods

This was a double-blind experimental investigation in which the sensory parameters were assessed by a convenience sample of 118 untrained tasters from a public healthy center who gave written informed consent to participate. This healthy center is responsible for the secondary care of an area with about 180,000 inhabitants, descendants of diverse ethnicities and coming from many regions of the country.The study was approved by the Ethics Committee of the Faculdade de Medicina de Ribeirão Preto - Universidade de São Paulo (Protocol no. 464/CEPCSE- FMRP-USP; 09/11/2011) and met the guidelines of the responsible governmental agency.

Exclusion criteria were: (1) food intolerance, (2) urinary incontinence (3), renal insufficiency, (4) presence of flu, colds, or any oral disease that would affect taste on the day of the experiment, (5) alcohol abuse (intake of more than 14 alcoholic drinks per week), (6) cognitive deficit (7), taking medications that might alter gustatory sensitivity such as chemotherapeutic drugs, penicillin, metronidazole, hydrochloride, amphotericin, nortriptyline, hydrochloride, carbamazepine, biguanide, etambutol, phenylbutazone, fluorouracil, allopurinol, penicillamine, or levodopa; (8) pregnancy (9), and having been submitted to radiotherapy of the head and/or cervical region.

After the exclusions, and factoring individuals who refused to participate, four groups of both genders were studied: 32 older hypertensive individuals aged 60 to 80 who were under treatment (OH), 28 older normotensive volunteers aged 60 to 80 years (ON), 25 young hypertensive subjects aged 30 to 50 years (YH), and 33 young normotensive subjects aged 30 to 50 years (YN). The experiments was conducted over 10 consectives months.

### Procedures

The general data of each volunteer were obtained using a semistructured questionnaire, including previous diagnoses, use of medications, smoking status, and alcohol consumption. Weight and height were measured and BMI was calculated for all participants.

As a reference of daily sodium and potassium intake, 24- hour urinary sodium and potassium excretion was determined on each of the 2 days preceding the experiment.^21^ Urine collection started with voiding and discarding the first urine in the morning after waking up. Subsequently, the urine excreted during the next 24 hours, up to and including the first voiding of the following day, was collected. A second 24-hour urine collection was performed 2 weeks later before the second experiment, for futher determination of sodium and potassium excretion.

Blood pressure was measured with a semiautomatic instrument (Omron HEM-431 CINT), with 3 measurements on the upper right limb and 3 measurements on the upper left limb after the patients rested in the sitting position for 5 minutes. The measurements were repeated after 2 weeks.

On the first day of the experiment, 3 samples of french bread rolls of the same composition except for different amounts of salt were prepared. Salt (1.4%, 2.0%, and 2.7%) was added to each kg. French bread habitually sold in this community contains, on average, 2% salt in its composition. Therefore, we provided a sample of bread with less salt (1.5% salt; 30% less salt than ordinary bread), a sample with the usual percentage of salt (2.0%), and a sample of bread with higher salt content (2.7% salt; 30% more salt than usual bread).

The three bread samples were prepared on the day of the test and offered to the volunteers in a random manner in disposable paper bags coded with random 3-digit numbers so that the investigator involved in the test would be unaware of the salt content of each sample. For the tasting, the samples were tested from left to right with a standard size of 10 to 15 g each in order to provide uniformity. The patients drank mineral water at room temperature between samples in order to help remove the taste. At the end of the test, the volunteers, who did not know that the bread samples contained different amounts of salt, were asked to state which sample they preferred. The participants were asked to avoid eating and drinking 2 h before the experiment.

In the second experiment, two weeks later, the participants were asked to again taste the 3 samples of french bread containing the same different amounts of salt as in the first experiment (1.5%, 2.0% and 2.7%), but now also containing oregano as an added spice (0.23 g/100 g of bread) and to state their preference. There were no changes in medication between the first and the second tests.

### Data analysis

Firstly, an exploratory analysis of the data was performed. Continuous variables with normal distribution are reported as mean + standard deviation and categorical variables are presented as absolute numbers and percentages. The Fisher exact test was used to compare categorical variables, the McNamer's test was used to evaluate the effect of the intervention and ANCOVA was proposed to compare the groups and to verify the effect of the covariates.^22^ This analysis assumes that its residues have a normal distribution with mean 0 and variance s^2^ constant. Transforms were used in response variables that did not reach the assumption. Differences were considered to be statistically significant when p < 0.05. The SAS system (version 9; SAS Institute, Cary, NC) was used for all statistical calculations.

## Results

Gender and alcohol consumption distribution was similar in all groups (p = 0.63; p = 0.26). There was a higher percentage of smokers among young patients than elderly ones (p < 0.001) (Table 1).

**Table 1:** Characteristics and Distribution of clinical data of the volunteers included in the study

	Young Hypertensive Subjects (YH); n = 25	Young Normotensive (Y) Subjects; n = 33	Older Hypertensive Subjects (OH); n = 32	Older Normotensive Subjects (ON); n = 28	P Value
**Sex**					**p = 0.63**
Male	11(44.0%)	13 (30.3%)	9 (28.1%)	10 (35.7%)	
Female	14(56.0%)	20 (60.7%)	23 (71.9%)	18 (64.3%)	
**Use of Alcohol**					**p = 0.26**
Yes	9 (36.0%)	15 (45.5%)	8 (25.0%)	7 (25.0%)	
No	26 (64.0%)	18 (54.5%)	24 (75.0%)	21 (75.0%)	
**Smoking Habit**					**p < 0.001**
Yes	6 (24.0%)	4 (12.2%)	0(0%)	1(3.5%)	
No	19 (76.0%)	29 (87.8%)	32 (100%)	27 (96.5%)	
Age, years	40.8 ± 6.2	35.6 ± 4.4	73.6 ± 6.3	71.4 ± 7.8	
SBP, mmHg	137 ± 15	116 ± 11	134 ± 16	125 ± 12	p < 0.05*
DBP, mmHg	86 ± 9	75 ± 9	79 ± 9	75 ± 7	p < 0.05**
UNaV, mEq/L	181.0 ± 74.2	127.6 ± 36	177.3 ± 62.3	129.6 ± 3 6.6	p < 0.05*
UKV, mEq/L	46.2 ± 13.4	40.3 ± 12.8	45.2 ± 14.5	35.2 ± 10.1	p < 0.05***
BMI, kg/m^2^	29.1 ± 5.0	25.9 ± 4.0	29.1 ± 5.1	27.0 ± 4.0	p = 0.02*

SBP: systolic blood pressure; DBP: diastolic blood pressure; BMI: body mass index; UNaV: 24-hour urinary sodium excretion; UKV: 24-hour urinary sodium excretion; p*: hypertensive groups vs. normotensive groups; p**: young hypertensive group vs. other groups.

Urinary sodium excretion was higher in the hypertensive groups (young and older subjects) than in the normotensive groups (young and older volunteers) (p < 0.05) (Table 1), and was higher in men than in women (men: 170.9 ± 73.6 mEq/24 h vs. women: 142.6 ± 46.0 mEq/24 h, p = 0.01). Volunteers who consumed alcohol had higher sodium excretion than those who did not (p = 0.02). Mean urinary potassium excretion was lower in normotensive elderly subjects than in the other groups (p < 0.05) (Table 1). Systolic BP was higher in the hypertensive groups (young and older subjects) compared to the non-hypertensive groups (young and older subjects), with p < 0.05. Mean diastolic BP was significantly higher in the group of YH subjects compared to all other groups of volunteers (p < 0.05) (Table 1).

BMI was higher in the hypertensive groups than in the normotensive ones (p = 0.02) (Table 1).

On both days of the experiment, men preferred the saltier samples compared to women (p < 0.01 in the first experiment and p = 0.01 in the second one).

Alcohol consuming volunteers preferred more often the saltier samples in the first experiment than the other volunteers (p = 0.04) but this difference no longer existed when oregano was added to the samples (second experiment) (p = 0.10). No difference was observed between smokers and nonsmokers.

On the two days of the experiment, gender, alcohol consumption and smoking did not show any differences in the elderly groups (hypertensive and normotensive), whereas differences were observed between the younger groups. In the first experiment, women in the YN group preferred more often samples with less salt, while men preferred more often saltier samples (p < 0.01). In the second experiment (oregano addition), the distribution was similar between genders, with a nonsignificant trend to a change in preference for less salty samples among men (p = 0.06). In the YH group, those who consumed alcohol in the first experiment more often preferred the saltier samples, and in second experiment (oregano addition), they began to prefer less salty bread samples (p = 0.04).

On the first day of the experiment, there was a different preference between the elderly hypertensive and normotensive groups (p < 0.01), with the elderly hypertensive group showing a greater preference for saltier samples which persisted in the second experiment (p = 0.02).

The YH group showed greater preference for samples with higher salt concentrations compared to YN subjects (p = 0.02), with this difference persisting in the second experiment (p < 0.01). The groups of elderly and YH had a similar distribution in the first and second experiment (p = 0.27 and p = 0.25), and the elderly and YN groups did not differ in preference (p = 0.11 and p = 0.34).

**Figure 1:**
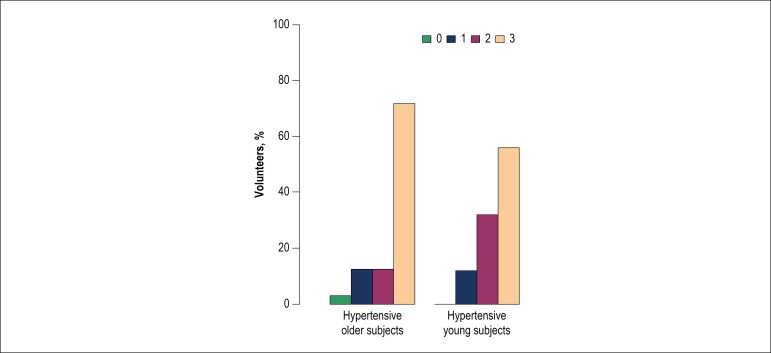
Distribution of the preference for bread samples among hypertensive volunteers in the first experiment, without the addition of oregano. 0: did not perceive a difference; 1: preferred the sample with 1.5% salt; 2: preferred the sample with 2.0% salt; 3: preferred the sample with 2.7% salt.

**Figure 2:**
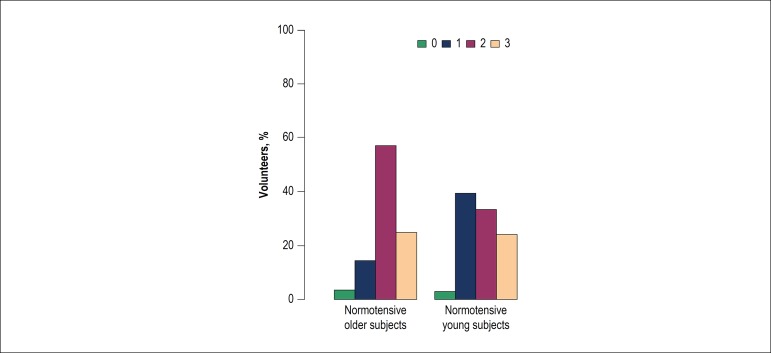
Distribution of the preference for bread samples among normotensive volunteers in the first experiment, without the addition of oregano. 0: did not perceive a difference; 1: preferred the sample with 1.5% salt; 2: preferred the sample with 2.0% salt; 3: preferred the sample with 2.7% salt.

**Figure 3:**
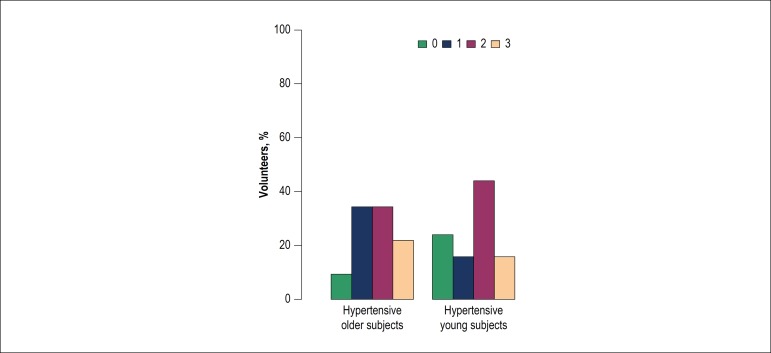
Distribution of the preference for bread samples among hypertensive volunteers in the second experiment, with the addition of oregano. 0: did not perceive a difference; 1: preferred the sample with 1.5% salt; 2: preferred the sample with 2.0% salt; 3: preferred the sample with 2.7% salt.

**Figure 4:**
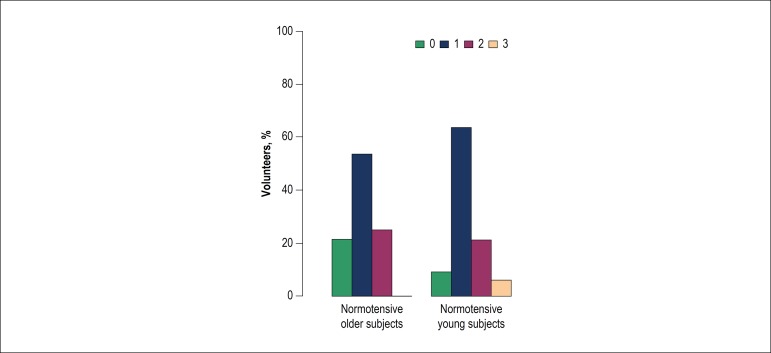
Distribution of the preference for bread samples among normotensive volunteers in the second experiment, with the addition of oregano.0: did not perceive a difference; 1: preferred the sample with 1.5% salt; 2: preferred the sample with 2.0% salt; 3: preferred the sample with 2.7% salt.

Comparing the first and second experiments, there were significant changes in all groups. In the hypertensive groups (young and older subjects), there was a predominance of preference for saltier samples in the first experiment (Figure 1), whereas a change in preference for standard bread samples and samples with a lower salt concentration (p < 0.01 for the hypertensive elderly group and 0.04 for the YH group) was observed in the second experiment (Figure 2). In the group of ON volunteers, in the first experiment the preference was more common for standard bread samples (Figure 3), whereas a greater preference for the sample with a lower salt concentration as observed in the second experiment (with the addition of oregano) (p < 0.01) (Figure 4). In the first experiment, YN subjects showed a higher preference for the samples with lower salt concentrations (Figure 3), with an increase in this preference in the second experiment (p = 0.03) (Figure 4). Oregano seems to contribute to the failure to differentiate flavors, with the total number of volunteers who did not notice a difference between samples increasing from 3 (3.13%) to 18 (15.25%), with p < 0.001.

## Discussion

We found a clear predominance of salt preference in hypertensive participants compared to the non-hypertensive groups. In this study, we used a sensory analysis to investigate salt preference using a method similar to that employed by Shepherd et al.,^23^ who compared the preference of hypertensive individuals for soup samples with three different salt concentrations. In the cited study, there was a greater preference for the sample with lower salt concentration, in contrast to what was observed in the present study. However, all of the participants in the Shepherd study were hypertensive, with no comparison with normotensive subjects.

The preference for high salt intake can be caused by physiological, genetic and psychological factors and by changes occurring during human development.^24^ Evidence indicates that the taste of salt is inherently attractive to humans by making the flavor of the foods more palatable compared to the same foods without salt.^24,25^

Regarding age, there were no differences in preference for salt samples and salt intake. The same result was reported by Khadeja & Leshen,^26^ who compared salt appetite in the elderly (65-85) and in middle-aged (45-58) people to determine possible age-related changes. To estimate salt appetite, the participants were tested for preferred amounts of salt in the soup, followed by a test with oral sprays of NaCl. Between the taste tests, the participants were interviewed to complete a dietary, seasoning and preference questionnaire. The authors found no clear difference in salt preference in elderly participants compared to middle-aged participants.

Regarding gender, men usually prefer more salt than women.^27^ It is well established that salt intake is lower in women as a function of their lower absolute caloric intake and that women add less salt to soups than men.^27^ The present study also showed a higher salt consumption among men.

The variables that significantly influenced the preference for samples of saltier breads were the presence of hypertension, male gender and alcohol consumption. These were maintained even in the presence of oregano, except for subjects with higher alcohol consumption, for which the preference for saltier samples ceased to exist when oregano was added to bread.

Contrary to our hypothesis, we found no relationship between sodium intake and age, BMI or tobacco use, but sodium intake was significantly higher among volunteers who consumed alcohol. These volunteers preferred saltier samples and showed an average larger amount of sodium excretion in 24hour urine, in agreement with data reported by Gibson & Margaret^28^ who showed that high alcohol intake is one of the major features among high salt consumers. It is important to note that one of the exclusion criteria for the present study was alcohol abuse so that the effect of alcohol on salt preference could have been underestimated if individuals with greater consumption had been included. Alcohol consumption has been associated with increased BP and an increased risk of hypertension in many observational studies and clinical trials, demonstrating that these associations are causal.^29^ Hypertensive volunteers had higher mean BP and higher average urinary sodium excretion compared to normotensive volunteers, in agreement with the association between increased sodium excretion and increased arterial BP reported in several studies.^19,20,30,31^ Thus, it was observed that hypertensive patients had higher mean BP even when treated, with a greater preference for salt intake and with higher urinary sodium excretion compared to normotensive subjects.

These results support recommendations for the reduction of high salt intake in the population for prevention and control of adverse BP levels.^20^

Despite the small percentage of smokers included in this study (9.3%), no significant differences were identified as to their preferences. Recent research in Germany has shown that smoking did not present a risk to gustatory commitment, although food preferences were not compared and the average age of individuals who participated in the study was 56 years.^32^

For potassium intake, the present study found increased urinary potassium excretion in the hypertensive groups, in contrast to the study of Galletti et al.,^33^ which showed low potassium excretion in 24hour urine in 1232 hypertensive Italians from 47 volunteer centers of the Italian Society of Hypertension. Observational studies have shown an inverse relationship between potassium intake and BP.^19^ The electrolyte excretion in 24-hour urine analysis and BP in the INTERSALT study^19^ showed that potassium excretion was negatively correlated with BP. Cappuccio et al.^34^ performed a meta-analysis of 19 studies with oral potassium supplementation involving 586 participants. The results showed that oral potassium supplementation significantly reduced both systolic and diastolic BP and that reductions in BP were higher in hypertensive patients than in normotensive individuals. Perhaps the finding in the present investigation differing from other studies could be explained by the fact that the volunteers had been regularly monitored at a secondary level health center for many years, having received nutritional guidance regarding a balanced diet rich in fruits and vegetables, and greater potassium intake.

It is important to emphasize that this study did not evaluate long-term adherence to bread with oregano, only preference in a tasting test in a small sample. A larger, longer, and randomized clinical trial is needed to confirm the benefits of an intervention contributing to the reduction of daily sodium intake, by adding spice to food.

The volunteers were followed up at a public health center, not being able to extrapolate the results to different populations.

## Conclusions

The present study demonstrated a greater preference for salt and more salt consumption in hypertensive than normotensive individuals regardless of age. The intervention of adding oregano to food led to a preference for samples with lower salt content in all groups, i.e., hypertensive, normotensive, young or old subjects. A higher preference for salt was found to be associated with male gender and alcohol consumption.
